# Prevalence of *Plasmodium falciparum* and non-*P. falciparum* infections in a highland district in Ghana, and the influence of HIV and sickle cell disease

**DOI:** 10.1186/s12936-017-1823-y

**Published:** 2017-04-24

**Authors:** Ewurama D. A. Owusu, Charles A. Brown, Martin P. Grobusch, Petra Mens

**Affiliations:** 1 Department of Medical Laboratory Sciences, School of Biomedical and Allied Health Sciences, College of Health Sciences, University of Ghana, Korle-bu, Accra, Ghana; 2 Center of Tropical Medicine and Travel Medicine, Department of Infectious Diseases, Division of Internal Medicine, Academic Medical Centre, University of Amsterdam, Amsterdam, The Netherlands; 3 Department of Medical Microbiology, Clinical Parasitology, Division of Laboratory Specialisms, Academic Medical Centre, University of Amsterdam, Amsterdam, The Netherlands; 4 Centre de Recherches Médicales de Lambaréné (CERMEL), Hôpital Albert Schweitzer, Lambaréné, Gabon; 5 Institute of Tropical Medicine, University of Tübingen, Tübingen, Germany

**Keywords:** Falciparum, Non-falciparum, Malaria, Ghana, HIV, SCD, Highland

## Abstract

**Background:**

In the past two decades, there has been a reported decline in malaria in Ghana and the rest of the world; yet it remains the number one cause of mortality and morbidity. Human immuno-deficiency virus (HIV) and sickle cell disease (SCD) share a common geographical space with malaria in sub-Saharan Africa and an interaction between these three conditions has been suggested. This study determined the *Plasmodium falciparum* and non-*P. falciparum* status of symptomatic and non-symptomatic residents of Mpraeso in the highlands of Kwahu-South district of Ghana based on evidence of current national decline. The influence of HIV and SCD on malaria was also determined.

**Methods:**

Participants were 354 symptomatic patients visiting the Kwahu Government Hospital and 360 asymptomatic residents of the district capital. This cross-sectional study was conducted during the minor rainy season (October–December 2014). Rapid diagnostic tests (RDT), blood film microscopy and real-time polymerase chain reaction assessment of blood were done. Participants who tested positive with RDT were treated with artemisinin-based combination therapy; and assessment of venous blood was repeated 7 days after treatment. HIV screening and haemoglobin genotyping was done. Univariate and multivariate regression analysis was used to determine the influence of SCD and HIV.

**Results:**

*Plasmodium falciparum* was prevalent at 124/142 (87.3%). *Plasmodium malariae* was the only non-falciparum species detected at 18/142 (12.7%). HIV and SCD did not significantly increase odds of malaria infection. However, the use of ITN and recent anti-malarial intake significantly decreased the odds of being malaria infected by 0.45-fold and 0.46-fold respectively.

**Conclusion:**

*Plasmodium falciparum* and *P. malariae* infection are the prevailing species in the study area; albeit varying from the national average. HIV and SCD were not associated with the risk of having malaria.

**Electronic supplementary material:**

The online version of this article (doi:10.1186/s12936-017-1823-y) contains supplementary material, which is available to authorized users.

## Background

Current indications are that malaria is on the decline in Ghana and other countries [1–5]. This might be due to various policies that have been implemented. For instance in Ghana, the adoption of intermittent preventive treatment in pregnant women with sulfadoxine–pyrimethamine (IPTp-SP) has been shown to substantially reduce placental malaria in women in the southern part of Ghana [1]. Another reason is the countrywide free distribution of insecticide-treated bed nets (ITN) [2, 6, 7]. Also in recent years the national policy has shifted from the presumptive approach to treatment (where patients with clinical symptoms are treated with anti-malarials) to test-based approach, where parasitological confirmation is established with microscopy, rapid diagnostic tests (RDT) or polymerase chain reaction (PCR) [2, 4, 8].

This significant decrease in prevalence of malaria in Ghana may result in marked changes in the current species dominance. Of the five *Plasmodium* species currently known to infect humans—*Plasmodium falciparum*, *Plasmodium ovale*, *Plasmodium malariae*, *Plasmodium vivax* and *Plasmodium knowlesi*—only the first three are documented to occur in Ghana [3]. *Plasmodium falciparum* is the prevailing species (90–98%), with few *P. malariae* (2–9%) and *P. ovale* (1%) [4], infections found. Subspecies *P. ovale wallikeri* (variant type) and *P. ovale curtisi* (classic type), which have been identified across sub-Saharan Africa [5–8] and Asia [9], have also been observed in Northern Ghana [10].

The malaria hypothesis has shown that sickle cell disease (SCD) and malaria share a common geographical space [11]. Ghana, a malaria hyperendemic country, has about 18.8% of its population possessing the haemoglobin S (HbS) allele [12]. Human immunodeficiency virus (HIV) and SCD incidentally share the same geographical area; interactions between them have been observed [13]. On the one-hand, HIV has been shown to increase the susceptibility of individuals to malaria and the severity of malaria [14, 15]. However, severity is increased in people who do not have prior acquired partial immunity to malaria [14, 15]. The performance of anti-malarials in people living with HIV have also been shown to be reduced [14]. On the other hand, it is well known that SCD influences malaria; HbAS has been shown to confer partial malaria immunity on individuals living in malaria endemic areas [16, 17]. In contrast, homozygous HbC has been suggested to increase the risk of infection with *P. malariae* [16]. However, the influence of HbSS on increased susceptibility to malaria is unclear [16, 18]. The authors postulate that in this study setting, where all three diseases overlap, SCD and HIV may influence infection with *Plasmodium* spp. The aim of this study was, therefore, to evaluate the state of *P. falciparum* and non-*P. falciparum* infections in a highland in the Eastern region of Ghana; and to determine the possible influence of SCD and HIV.

## Methods

### Study site and population

Ghana is divided into three malaria epidemiological zones; namely, northern savannah, tropical rainforest and coastal savannah and mangrove swamps [4]. The study site, Kwahu-South, is located in the tropical rainforest zone. Malaria occurs all year round, and is influenced by precipitation [19]. There are two rainy seasons in Ghana: the major rainy season (from May to June) and minor rainy season (from September to October) [19, 20].

The first group of study participants was recruited from the Kwahu Government Hospital, which is one of two district hospitals that serve communities in the entire Kwahu mountain ridge and beyond [21]. An estimated population of 230,000 people from more than 200 communities in the area of 1876 sq km receive services from this hospital [22]. The hospital has 175 beds and provides 24-hour services [22]. The Outpatient Department (OPD) serves about 250 patients daily, who are subsequently referred to the appropriate specialist department [22]. It also runs an anti-retroviral therapy (ART) clinic that attends to the health and therapeutic needs of patients living with HIV. About 65,000 confirmed malaria cases are reported in the district annually [21]. The second group of participants was recruited from the community; they were residents of the Kwahu-South district capital. Description of residents in the Kwahu-South district capital has been reported in detail elsewhere [23].

In Ghana, anyone presenting at the health facililty with axillary temperature ≥37.5 °C without any other symptom is suspected of malaria [24]. The current management is to first diagnose with RDT or microscopy and then treat with anti-malarial when positive [24]. Parasitological confirmation, in the absence of signs of severe disease is classified as uncomplicated malaria [24, 25]. Signs of severe/complicated malaria include unconsciousness, marked jaundice and difficulty breathing [24, 25]. The national policy is to use oral artemisinin-based combination therapy to treat uncomplicated malaria [24]. First line drugs for uncomplicated *P. falciparum* infections are artesunate–amodiaquine (AS+AQ), artemether–lumefantrine (A–L) and dihydroartemisinin–piperaquine (DHAP); the second line drug is oral quinine (QN) for 7 days [4, 24]. Intra-venous or intra-muscular anti-malarials are encouraged when severe malaria is identified, whilst waiting for laboratory results [24].

### Study design and participant selection

This was a cross-sectional study, done during the minor rainy season, from October to December in 2014. Patients presenting at the OPD with an axillary temperature ≥37.5 °C with or without vomiting, nausea, general malaise, headaches or body aches were recruited into this study and classified as symptomatic. There was no age or sex discrimination in participant selection. Demographic data and medical history of participants were collected by trained collectors prior to sample collection, and after obtaining written or finger-stamped informed consent. Demographic data included age, sex and education whilst medical history included axillary temperature, symptoms, diabetes status and recent anti-malarials taken. In instances where participants could neither read nor write, questions were asked in the local language and their responses were filled in by the trained sample collectors. For children under the age of 18 years, their parents/guardians provided responses on their behalf.

### Laboratory procedures

Expert phlebotomists took venous blood from the antecubital fossa of participating patients at the hospital. This was stored in ethylenediaminetetraacetic acid (EDTA) tubes at 4 °C until processed. Participants from the community had their finger-prick capillary blood taken. Thick and thin blood smears were prepared from all samples. After air-drying, thin smears were fixed in 100% methanol. All smears were stained with 10% Giemsa which was applied to both thick and thin films for 10 min. These were then examined by two expert microscopists. Samples were recorded as positive under 200 high powered visual fields if any amount of asexual parasites was observed per 200 white blood cells, and negative if none was observed. Discordant results were decided on by a third microscopist. A drop of each venous blood sample taken was applied to the malaria RDT First Response^®^ Malaria Ag. *Plasmodium falciparum* (HRP2), to test for the presence of *P. falciparum.* Patients who tested positive for malaria, with either RDT or microscopy, were prescribed the first-line artemisinin-based combination therapy (ACT) artemether–lumefantrine by the attending physician, according to the national malaria control programme. It is a fixed-dose formulation of 20 mg artemether and 120 mg lumefantrine per tablet [3]. This is a 6-dose regimen taken over 3 days according to pre-defined weight bands [17]. Patients who had malaria were asked to return to the hospital 7 and 28 days after ACT treatment for re-testing with RDT and microscopy. Standard Hb genotyping [26] was done with about 1 ml whole blood and categorized into HbAA (normal), HbAS (trait), HbAC (trait), HbSS (SCD) or HbSC (SCD).

Two to three drops of each blood sample was spotted onto Whatman filter paper (grade 3), dried and stored and PCR analysis done at KIT Biomedical Research Institute in Amsterdam. Boom extraction, as previously described [27], was used to obtain DNA. Real time PCR of DNA was conducted on Biorad C1000™ Thermal Cycler to identify pan-*Plasmodium*. Species identification was done, on positive samples, with nested PCR amplification of small sub-unit ribosomal RNA genes as previously described [28]. Gel electrophoresis was used to resolve PCR products with 2% agarose. The second round species-specific primers included that for detection of *P. ovale wallikeri* and *P. ovale curtisi* as described [29].

Blood samples of hospital participants were further tested for HIV using INSTANTCHEK HIV-1 + 2 rapid diagnostic test kit by EY Laboratories and Oraquick Advance Rapid HIV-1/2 antibody test by OraSure Technologies, Inc. The use of Oraquick Advance Rapid HIV-1/2 antibody test is consistent with the confirmatory testing protocol for HIV in Ghana [30].

### Statistical analyses

Data was entered into Microsoft Excel and then transferred into IBM SPSS version 20 software (IBM inc, Chicago, IL, USA) for statistical analyses. Univariate and multivariate regression analysis were used in determining whether primary outcome of malaria positivity was influenced by several independent variables, after adjusting for confounders. Independent variables included sex, sickle cell status, HIV, diabetic status and anti-malarial history. The strength of association was measured by odds-ratio with a confidence interval of 95%. *P* value ≤0.05 was regarded as statistically significant.

## Results

The baseline medical and demographic history of participants from the OPD and community is summarized in Table 1. In general, malaria prevalence amongst participants in the hospital was 93/354 (26.3%); and 43/360 (11.9%) amongst asymptomatic residents, when microscopy was used. Females in this study were 314/354 (88.7%) and 164/360 (45.6%) in the hospital and community, respectively. Participants from the community were equally distributed amongst the age groups. In the hospital, the majority of participants (155/354; 43.8%) was 19–30 years old. Majority of participants, 250/354 (70.6%) in symptomatics and 218/360 (60.5%) in asymptomatics had not taken any anti-malarials in the past 6 months, according to their responses in the questionnaire. About 25% (92/354) of symptomatic patients had a haemoglobinopathy of some sort; 41/354 (11.6%) having HbAC and 45/354 (12.7%) having HbAS. On the other hand, 84/360 (23.3%) of asymptomatics had HbS or C. The HIV prevalence in the hospital participants was 51/354 (14.4%) with 9/354 (2.5%) participants testing positive for both HIV 1 and 2.

**Table 1:** Baseline characteristics of study participants in the district capital and the Kwahu Government Hospital

Variable	n (%)	
Sex	Hospital	Community
M	40 (11.3)	196 (54.4)
F	314 (88.7)	164 (45.6)
Age group (years)		
≤5	23 (6.5)	60 (16.7)
6–10	12 (3.4)	60 (16.7)
11–18	39 (11)	60 (16.7)
19–30	155 (43.8)	60 (16.7)
31–60	105 (29.7)	60 (16.7)
Over 60	20 (5.6)	60 (16.7)
Recent anti-malarial intake		
<3 months	29 (8.2)	55 (15.3)
3–6 months	75 (21.2)	87 (24.2)
No	250 (70.6)	218 (60.5)
Preventive measures		
None	16 (4.5)	142 (39.4)
ITN	58 (16.4)	104 28.9)
Other (mosquito spray & coil)	280 (79.1)	114 (31.7)
Sickling status		
HbAA	262 (74)	276 (76.7)
HbAC	41 (11.6)	30 (8.3)
HbAS	45 (12.7)	43 (11.9)
HbSC	4 (1.1)	6 (1.7)
HbSS	2 (0.6)	5 (1.4)
HIV status		
Neg	299 (85.6)	
HIV 1	42 (11.9)	
HIV 2	0 (0)	
HIV 1 & 2	9 (2.5)	
Malaria^a^		
Negative	261 (73.7)	317 (88.1)
Positive	93 (26.3)	43 (11.9)
Other conditions		
Diabetes	13 (3.7)	
Pregnancy	94 (26.6)	

^a^Malaria was diagnosed with microscopy

Overall, gold standard microscopy showed falciparum infections were 124/142 (87.3%) whilst non-falciparum infections were 18/142 (12.7%) (Table 2). Amongst the 49 participants who had non-falciparum malaria, 9 (9.7%) were asymptomatic; only *P. malariae* was present (Table 2). No patient tested positive for *P. ovale curtisi*, *P. ovale wallikeri* or *P. vivax*. In general, malaria was prevalent amongst participants with the normal HbAA allele. The normal Hb allele (AA) was found in 86/124 (69.4%) participants with falciparum malaria; and in 11/18 (61.1%) of participants with non-falciparum malaria. In both symptomatics and asymptomatics, malaria was prevalent in participants who had not taken anti-malarials recently (Table 2). Table 3 summarizes the factors that are independently associated with the risk of symptomatic *Plasmodium* infection. History of recent anti-malarials taken had a significant 0.46-fold decrease in odds of malaria for both univariate and multivariate); the use of ITN showed a significant 0.45-fold decrease in odds for malaria. Data and anyalysis for this study may be found in supplementary file (Additional file 1).

**Table 2:** Comparison of falciparum and non-falciparum malaria in asymptomatic and symptomatic participants, using the current gold standard in Ghana, microscopy

	Falciparum, n (%)		Non-falciparum, n (%)	
	Symptomatic	Asymptomatic	Symptomatic	Asymptomatic
	Day 1		Day 1	
*Plasmodium* spp.	84 (90.3)	40 (81.6)	*P. malariae*, 9 (9.7)	9 (18.4)
			*P. ovale curtisi*, 0 (0)	0 (0)
			*P. ovale wallikeri*, 0 (0)	0 (0)
			*P. vivax*, 0 (0)	0 (0)
Species dominance	124 (87.3)		18 (12.7)	
	Day 8		Day 8	
*Plasmodium* spp.	3 (3.2)		*P. malariae*, 7 (7.5)	

	Falciparum infection		Non-falciparum infection	
	Symptomatic	Asymptomatic	Symptomatic	Asymptomatic
Sex				
M	10 (11.9)	21 (52.5)	9 (100)	3 (33.3)
F	74 (88.1)	19 (47.5)	0 (0)	6 (66.7)
Age group ( years)				
≤5	3 (3.6)	7 (17.5)	0 (0)	1 (11.1)
6–10	4 (4.8)	9 (22.5)	0 (0)	0 (0)
11–18	17 (20.2)	10 (25)	4 (44.4)	4 (44.4)
19–30	37 (44.0)	6 (15)	5 (55.6)	0 (0)
31–60	20 (23.8)	5 (12.5)	0 (0)	1 (11.1)
Over 60	3 (3.6)	3 (7.5)	0 (0)	3 (33.3)
Sickling status				
Hb AA	64 (76.2)	22 (55)	5 (55.6)	6 (66.7)
Hb AS	9 (10.7)	12 (30)	2 (22.2)	0 (0)
Hb AC	10 (11.9)	6 (15)	2 (22.2)	3 (33.3)
Hb SS	1 (1.2)	0 (0)	0 (0)	0 (0)
Hb SC	0 (0)	0 (0)	0 (0)	0 (0)
Recent anti-malarial intake				
<3 months ago	5 (6)	3 (7.5)	1 (11.1)	3 (33.3)
3–6 months ago	11 (13.1)	7 (17.5)	2 (22.2)	2 (22.2)
None	68 (26.9)	30 (75)	6 (66.7)	4 (44.5)
HIV				
HIV 1	13 (15.5)	0 (0)	0 (0)	0 (0)
HIV 2	0 (0)	0 (0)	0 (0)	0 (0)
HIV 1 & 2	2 (2.2)	0 (0)	0 (0)	0 (0)
Negative	69 (82.1)	40 (100)	9 (100)	9 (100)
Diabetes				
Positive	3 (23.1)	0 (0)	0 (0)	0 (0)
Negative	81 (76.9)	40 (100)	9 (100)	9 (100)
Pregnancy				
Positive	17 (20.2)	0 (0)	4 (44.4)	0 (0)
Negative	67 (79.8)	40 (100)	5 (65.6)	9 (100)

**Table 3:** Univariate and multivariate regression analyses of predictor variables for *P. falciparum* and *P. malariae* infection

Variable	n (%)	Univariate analysis	Multivariate analysis
		Odds ratio (CI), P value	Odds ratio (CI), P value
*P. falciparum* positive	84 (23.7)		
SCD			
AA	262 (74)	1	1
AC	41 (11.6)	1.0 (0.46–2.15), 0.99	0.97 (0.45–0.10), 0.93
AS	45 (12.7)	0.77 (0.35–1.69), 0.52	0.70 (0.32–1.55), 0.38
SS	2 (0.6)	3.09 (0.19–50.17), 0.43	2.25 (0.11–45.85), 0.60
HIV			
Negative	299 (85.6)	1	1
HIV 1	42 (11.9)	1.49 (0.74–3.03), 0.27	1.33 (0.64–2.78), 0.45
HIV 1 & 2	9 (2.5)	0.95 (0.95–0.19), 0.95	0.93 (0.19–4.67), 0.93
Diabetes			
Negative	341 (96.3)	1	1
Positive	13 (3.7)	0.96 (0.26–3.58), 0.96	0.83 (0.22–3.15), 0.78
Recent anti-malarial intake			
None	250 (70.6)	1	1
3–6 months ago	75 (21.2)	0.46 (0.23-0.92), 0.03*	0.46 (0.23–0.92), 0.03*
<3 months ago	29 (8.2)	0.56 (0.21-1.52), 0.25	0.50 (0.18–1.44), 0.20
Preventive measures			
None	16 (4.5)	1	1
ITN	58 (16.4)	0.45 (0.27–0.99), 0.04*	0.45 (0.27–0.99), 0.04*
Other (mosquito spray & coil)	280 (79.1)	1.58 (0.25–5.48), 0.63	1.39 (0.17–5.23), 0.45
Sex			
F	314 (88.7)	1	1
M	40 (11.3)	1.08 (0.51–2.31), 0.84	1.23 (0.53–2.82), 0.63

* Statistically significant with P value ≤0.05

## Discussion

Malaria prevalence and determinants as well as prevailing species in this highland of Ghana varied from the published prevalence of malaria in that administrative region of Ghana. This might be because of the heterogeneous nature of malaria transmission in the country. The prevalence in this study was 3.2% lower than prevalence published in the national survey [31]. Only two *Plasmodium* spp. out of the known three prevailed in this study area. *P. ovale* which occurs in 0.15% of known infections in Ghana [24], and persists in the two forms (*P. ovale curtisi* and *P. ovale wallikeri*) in different parts of the country [10], was absent in this area. *P. vivax* was not detected in this area. HIV and SCD did not influence malaria in this particular study.

Unlike studies done elsewhere in the country [10, 32], there were no *P. ovale curtisi* or *P. ovale wallikeri* infections in this study. Both *P. ovale curtisi* and *P. ovale wallikeri* have been found to circulate in the population in Ashanti region [10]; whilst *P. ovale wallikeri* was diagnosed in a foreign visitor to the country who visited different regions in the country [32]. Even though the occurrence of falciparum malaria was slightly lower than the Ghana national prevalence of 90–98% [4], *P. malariae* in this study was much higher at 12.7% compared to that of the national prevalence of 2–9% [4]. These differences may be attributed to the altitude and climate of this forest ecological zone. In other African countries, increase in altitude has been known to reduce malaria prevalence [33, 34]. Malaria transmission in Ghana is heterogenous; previous studies have shown villages in close proximity in another highland area of Ghana to be extremely heterogenous [35]. In this study, however, malaria prevalence and transmission dynamics of neighbouring towns and villages were not available for comparison. Yet heterogeneity was evident when findings in this study were compared with national prevalence rates.

PCR testing of malaria positive patients 7 days post treatment indicated parasite clearance in 80 out of 93 samples. A previous study in that area showed residents combined anti-malarials with local herbal preparations when treating malaria [18], and this might be the reason for the 13 uncleared parasite samples in this study. Herbal medicine has been known to negatively influence malaria parasite clearance [30]. Even though uncleared parasites might not influence subsequent incidence of clinical episodes [30], they can contribute to parasite reservoirs which are very important in malaria transmission. The persistence of sub-microscopic parasites is therefore a cause for concern and warrants further investigation.

Univariate and multivariate regression analysis of the risk of having malaria in symptomatic participants showed that HIV status and sickling status did not cause any significant increase or decrease in risk, even though 14.4 and 17.7% of participants carried the HbS allele and had HIV respectively. In this study neither HbSS nor HbAC showed any influence on malaria; other studies have also been unable to show a clear relationship between them [16, 18, 36]. Whilst certain studies have shown decreased risk for malaria in people with HbSS [37, 38], others have shown no difference between HbSS allele and HbAA where malaria risk is concerned [16, 18, 36]. Also, heterozygous HbAC has not been found to influence malaria in some studies [16], yet some studies have shown malaria to be reduced in people with HbAC [23]. The risk for malaria in this study was also not influenced by HIV, unlike previous studies which have suggested HIV increases risk for malaria [14, 15]. However, malaria risk was reduced when participant-reported recent anti-malarials intake was 3–6 months prior to study. The use of ITN also significantly reduced odds of malaria by 0.46-fold and 0.45-fold respectively. This is in agreement with the current knowledge that the use of ITNs are highly effective at preventing mosquito bites much better than the mosquito coils and sprays [39, 40].

Despite the Ghana national policy on intermittent preventive treatment in pregnancy (IPTp), which advocates the administration of sulfadoxine-pyrimethamine at monthly intervals after 16 weeks [24], 22.3% of pregnant women (gestation > 20 weeks) included in this study had malaria. They constituted 22.6% of the malaria positives. More than half of the pregnant women with malaria (14/21) had not taken any anti-malarials in the last 6 months. This could be due to low or late attendance of antenatal clinics in rural areas [41, 42], regardless of provision of free medical care for pregnant women under the National Health Insurance Scheme [41]. Another possible reason is the availability of poor quality sulfadoxine–pyrimethamine; some SP drugs on the Ghanaian market have been found to be of low quality. A recent study found 2 out of 3 SP drugs given at hospitals in the Central region of Ghana to pregnant women failed the drug dissolution test, even though they contained the active ingredient [43]. This means that the amount of the drug that was able to dissolve within a particular period and be available for absorption in the body was very low; the drugs were therefore not effective [43]. Drop-out rates for IPTp in pregnant women in Ghana have been attributed to shortage of the drugs, unavailability of water to take the drugs with when they are dispensed, and the attitude of the provider [42]. All of these are possible reasons for the results in this study.

Nonetheless malaria and malaria-related deaths have declined in the past 6 years [1, 44–47]. This has been attributed to the intensified efforts to eradicate malaria through the Ghana National Malaria Control Programme.

This study had certain limitations. Firstly, the study sample was small and therefore may not have been adequately sized to accurately measure local *Plasmodium* species prevalence and HIV status of all participants. Participants in the hospitals were more willing to have their HIV status determined; no one in the community aspect of the study agreed to the test. Thus, the absence of statistical findings in this study may not necessarily mean the absence of parasites or correlations, and larger cohort studies are therefore needed. Secondly, even though majority of patients returned for the first follow-up, none returned for the second. This prevented information on subsequent parasite clearance from being obtained. Thirdly, this study was done during the minor rainy season. An extension of the study to include both major and minor rainy seasons would have provided a clearer interpretation of the influence of rainy seasons on malaria.

## Conclusion

*Plasmodium* spp. type and prevalence in this study area varies from the other regions in Ghana. The high prevalence of the HbS allele in the study population did not significantly influence malaria; neither did HIV. Nonetheless, further large-scale studies are necessary to identify possible influence of SCD and HIV as has been suggested to occur elsewhere.

## Additional file

**Additional file 1.** The data analysis of malaria symptomatic and asymptomatic participants in this study.
